# *Ganiopteris* gen. nov., an early Carboniferous seed plant with foliar-borne ovules and its evolutionary significance

**DOI:** 10.1093/nsr/nwag429

**Published:** 2026-07-10

**Authors:** De-Ming Wang, Yi Zhou, Jiang-Nan Yang, Peng Xu, Le Liu, Min Qin, Yue-Han Pan

**Affiliations:** Key Laboratory of Orogenic Belts and Crustal Evolution, Department of Geology, Peking University, China; School of Ecology, Sun Yat-sen University, China; Zhejiang Institute of Geosciences, China; Key Laboratory of Orogenic Belts and Crustal Evolution, Department of Geology, Peking University, China; School of Geoscience and Surveying Engineering, China University of Mining and Technology (Beijing), China; College of Life Sciences, Linyi University, China; Key Laboratory of Orogenic Belts and Crustal Evolution, Department of Geology, Peking University, China

## Abstract

This paper reports a new genus Ganiopteris that represents the earliest-known seed plants with ovules or seeds (fertilized ovules) attached to leaves (leaf laminae) from the early Carboniferous (∼360 million years ago) stratum in China. Ganiopteris may provide essential information on the origin of the glossopterids (Glossopteridales), an extinct group of gymnosperms that once dominated the Permian (300-250 million years ago) Gondwana Continent including today’s Southern Hemisphere continents and India.

Early seed plants from Late Devonian and early Carboniferous are characterized by terminal ovules on dichotomous and leafless axes [1,2]. These ovules themselves may have performed the photosynthetic function [1,3]. Seed plants with foliar-borne ovules (used here for ovules attached to leaf laminae or pinnules) had not appeared until late Carboniferous when they were represented by some seed ferns including callistophytes and peltasperms; seed ferns or pteridosperms with fern-like fronds and seed-bearing leaves/branches are extinct gymnosperms in Late Palaeozoic and Mesozoic [4]. The derivation of sporophylls of some groups of euphyllophytes is ideally explained by telome theory [5], but that of seed plants is poorly known. As a unique group of seed ferns, the glossopterids (Glossopteridales) dominated Permian Gondwana Continent including today’s Southern Hemisphere continents and India [6]. Regarding numerous types of ovulate organs of glossopterids, their megasporophyll with abaxial ovules is consistently connected to the petiole or lamina of a subtending vegetative leaf [4,7]. Glossopterids tentatively originated in Carboniferous [8], when diverse glossopterid palynomorphs were widely reported from Gondwana [9]. Nevertheless, the macrofossils of glossopterids have never been discovered before Permian.

From Lower Carboniferous Huashanling Formation in three localities of Jiangxi Province, China (Fig. S1), we now report a novel seed plant *Ganiopteris phylla* gen. et sp. nov. (Fig. 1; Figs S2–S12). It is a rare example of a whole fossil plant in fertile characters, although vegetative characters such as branches and leaves are unknown. Fertile integrity of *Ganiopteris* includes complete fertile organs (megasporophylls with abaxial ovules), multiple megasporophylls arranged into a strobilus and terminal position of a strobilus on axis. It increases the fertile diversity, uniqueness and complexity of coeval pteridosperms. *Ganiopteris* represents the oldest gymnosperms with ovules attached to leaf laminae and suggests vital involvement of leaves in photosynthetic physiology of ovules. This genus may provide essential information on the origin of seed plants (including the glossopterids) with foliar-borne ovules.

**Figure 1.**
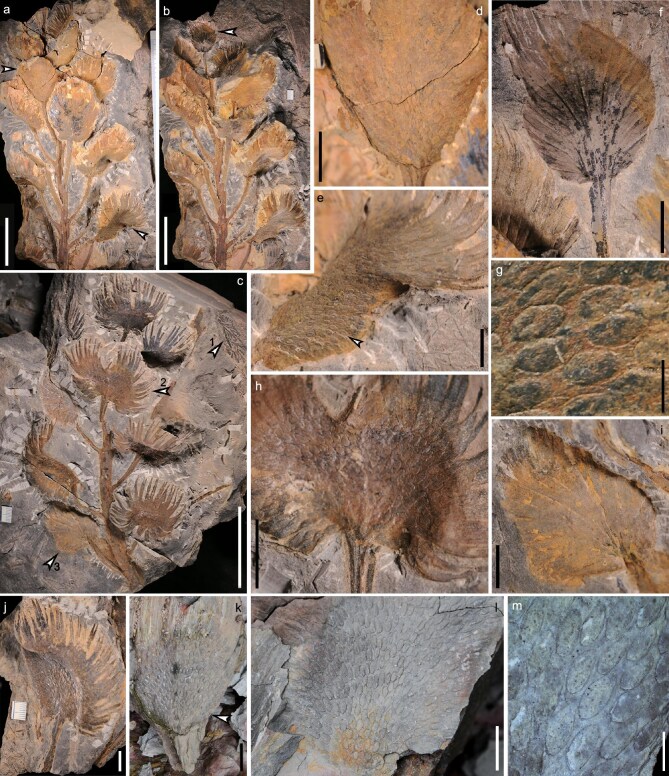
Strobili with megasporophylls of *Ganiopteris phylla* from the Lingtou section. (a) A strobilus with helically arranged megasporophylls. Upper and lower arrows indicate portions enlarged in panels (d) and (e), respectively (PKUB21721a). (b) Dégagement of mid-upper part of strobilus in panel (a). Arrow indicates the portion enlarged in panel (f). (c) A strobilus with helical sporophylls. Arrow 1 indicates associated vegetative foliage; arrows 2 and 3 indicate portions enlarged in panels (h) and (i), respectively (PKUB26002a). (d and e) Abaxial view of enlarged sporophylls from panel (a) (upper and lower arrows, respectively), with an arrow indicating the portion enlarged in panel (g). (f) Adaxial view of enlarged sporophyll from panel (b) (arrow). (g) Ovules on abaxial surface of sporophyll. (h and i) Abaxial and adaxial view of enlarged sporophylls from panel (c) (arrows 2 and 3, respectively). (j) Sporophyll in abaxial view (PKUB26001). (k and l) Sporophylls in abaxial view, with an arrow indicating the portion enlarged in panel (m) (PKUB26015, PKUB26016). (m) Ovules on abaxial surface of sporophyll. Scale bars: 5 cm (a–c), 1 cm (d–f and h–l) and 2 mm (g and m).

Twelve strobili are available (Fig. 1; Figs S2–S10). The ovuliferous fructifications (ovule-bearing organs) or megasporophylls are in loose helical arrangement (Fig. 1a–c; Figs S2a–d, S3a–d, S4a–d and f, S5a and b, S7a and b, S8a and b, S9a and b and S10a–c). Such an arrangement is recognized through serial dégagement of several fructifications, some of which have both the part and counterpart of a specimen. The long triangular scar left on strobilar axis (e.g. Fig. S2e, arrow, and f, arrow 3) corresponds to the base of a megasporophyll (pedicel) extending into rock matrix. One specimen shows that the strobilus

terminates an axis that is ca. 20 cm long and 2.0 cm wide (Fig. S4a–e). There are at least 20 megasporophylls preserved within a single strobilus (Fig. S2a–d). Megasporophylls are sometimes associated with vegetative foliage bearing *Rhodea/Rhodeopteridium*-like leaves (Fig. 1c, arrow 1; Fig. S6d). No broad-meshed forms of *Glossopteris* were discovered. The megasporophyll is up to 11.8 cm long (Fig. 1a), scutum-shaped and consists of a lamina with marginal wing-like lobes and a basal pedicel (Fig. 1f; Figs S4g, S6a and c–f and S8d). The lamina is obovate or flabellate in shape, with abaxial side bearing numerous ovules (Fig. 1d, e, h and j–l; Figs S2m, S3g, S4g, S5g, S6c–f, S7f and g, S8c and g, S10d and g–k and S11 and S12) and with adaxial side bearing distinct veins (Fig. 1f and i; Figs S2j–l, S6g–i, S8d–f and h–j and S9). The venation is radial in pattern and dichotomizes several times. The edges of megasporophyll lamina extend abaxially and then adaxially into many wing lobes (Fig. 1a–c, h and j; Figs S3a and e, S4f–h, S5a and d, S7 and S12a). Wing lobes appear distally perpendicular to lamina and sometimes dichotomize once. These lobes are elongate, distally tapered and seem to contain a single vascular strand (Figs S2g, S5f, S7c and h and S12b). The pedicel is connected to the base of lamina (Fig. 1f and h–l; Figs S2h–l, S3e–j, S4g, S6c–f and S8d and e) and occasionally shows a central vascular strand (Fig. S3h). Ovules appear flattened owing to preservation. They are usually ovoid in outline (Fig. 1g and m; Figs S2n and o, S5h–k, S10h, S11c–i and S12f–j). Ovules are densely arranged to be separated from one another by very little space. The micropyle is most probably present, since no integumentary lobes can be observed among multiple ovules. Other details of ovules are currently unknown.

Ovules (cupulate or acupulate, i.e. surrounded by cupules or not) of the earliest known seed plants in Late Devonian (Famennian) usually occur terminally or occasionally laterally on axes without pinnules [1,2]. Early Carboniferous (Mississippian) seed plants include calamopityaleans, buteoxylonaleans, lyginopteridaleans and medullosaleans [4]. Among them, the calamopityaleans and buteoxylonaleans are not exactly known for ovules. The lyginopteridaleans have ovules borne terminally on branches rather than attached to pinnules [10]. The medullosaleans have ovules variously arranged on frond axes [11]. Therefore, *Ganiopteris* now represents the oldest gymnosperms with foliar-borne ovules (ovules attached to leaf laminae). It indicates that the seed plants underwent the transition from axial- to foliar-borne ovules at the beginning of Carboniferous. Regarding Famennian seed plants, the axes bearing ovules lack laminate leaves or pinnules and thus the photosynthesis of ovules depends on cupules (if present) or on integumentary lobes [1,3]. Their individual fertile organs or fertile units refer to a single or several large ovules (up to 33 mm long and 5.7 mm wide) enclosed in a cupule or not [2]. In contrast, each fertile unit of *Ganiopteris* includes an enormous megasporophyll with numerous small ovules (ca. 2.6 mm long and 1.4 mm wide). Consequently, the photosynthetic function of ovules is largely and collectively performed by laminate leaves, and this plant physiology is more efficient.

Telome theory is successful in interpreting the derivation of laminate fertile leaves (sporophylls or fertile pinnules) in both sphenopsids and ferns from three-dimensional fertile branches bearing terminal sporangia [5]. This theory can also be used to explain the formation of sporophylls of seed plants, which is through planation and then lamination of ultimate branches terminated by ovules. In this scenario, some Famennian acupulate ovules borne axially, e.g. *Alasemenia, Guazia* and *Sinolobotheca* from South China Block [2], act as appropriate candidates for evolving foliar-borne ovules. Both cupulate and acupulate ovules existed in Carboniferous [4]. Therefore, we suggest that cupulate and some acupulate ovules extended from Late Devonian to Carboniferous and remained axial-borne. Famennian seed plants were suggested to possess laminate vegetative leaves/pinnules (e.g. *Elkinsia*) or planate leaf precursors (e.g. *Cosmosperma*) [12,13]. If so, in seed plants, sporophylls generally occur later than vegetative pinnules. Vegetative pinnules were widely documented [14], whereas sporophylls were rarely reported for Late Devonian sphenopsids. Vegetative pinnules and sporophylls characterize the ferns that first appeared in the earliest Carboniferous [15]. Vegetative pinnules were rare and sporophylls were absent in Middle Devonian–Carboniferous fern-like plants [16]. Vegetative pinnules and sporophylls are typical of some taxa of Devonian progymnosperms (Archaeopteridales) [4]. In general, laminate vegetative leaves independently evolved in these non-lycopsid vascular plants (euphyllophytes); laminate fertile leaves failed in fern-like plants, occurred approximately simultaneously in seed plants and ferns but later than in sphenopsids and progymnosperms.

After glacier retreat of Late Paleozoic Ice Age, Permian glossopterids dominated temperate ecosystems of high latitudinal Gondwana in Southern Hemisphere [6,7]. The hiatus below Lower Permian glacial deposits prevents the search for glossopterid ancestors [8], while Carboniferous palynomorphs from many Gondwanan localities clearly indicate the occurrence of macro fossils of glossopterids in this period [9]. During Carboniferous, South China was situated in Southern Hemisphere, near the eastern part of Gondwana, and within a tropical climate zone [17]. If *Ganiopteris* from South China represents a potential precursor/ancestral form, the origin of glossopterids could be traced to early Carboniferous when they were adapted to tropical climate. Laminate vegetative leaf subtending a polysperm functions in protecting glossopterid ovules [7]. Without this subtending leaf, the densely arrayed ovules of *Ganiopteris* could be protected by the block of neighboring laminae of megasporophylls in a strobilus. Unlike glossopterids, *Ganiopteris* may not necessarily need a subtending vegetative leaf to prevent the ovules from herbivory and desiccation. During early Carboniferous, (herbivorous) insects and vertebrates were so extremely rare as to leave the Hexapoda gap and a Romer’s gap [18]. In this period, South China was located within the tropical climate zone, probably receiving abundant rainfall [17].

## Supplementary Material

nwag429_Supplemental_File
