# Supplementary material for: Ganiopteris gen. nov., an early Carboniferous seed plant with foliar-borne ovules and its evolutionary significance
Source: Natl Sci Rev. 2026 Jul 10;13(15):nwag429. doi: 10.1093/nsr/nwag429 (PMC13455625; doi:10.1093/nsr/nwag429)
Supplement: Supplementary file 1 [file nwag429_supplemental_file.docx]

**Supplementary Data**

***Ganiopteris* gen. nov., an early Carboniferous seed plant with foliar-borne ovules and its evolutionary significance**

De-Ming Wang^1,^ *, Yi Zhou^2^, Jiang-Nan Yang^3^, Peng Xu^1^, Le Liu^4^, Min Qin^5^, Yue-Han Pan^1^

^1^Key Laboratory of Orogenic Belts and Crustal Evolution, Department of Geology, Peking University, Beijing 100871, China.

^2^School of Ecology, Sun Yat-sen University, Guangzhou 510275, China.

^3^Zhejiang Institute of Geosciences, Hangzhou 311202, China.

^4^School of Geoscience and Surveying Engineering, China University of Mining and Technology (Beijing), Beijing 100083, China.

^5^College of Life Sciences, Linyi University, Linyi 276000, China.

*Corresponding author. Email: dmwang@pku.edu.cn

Supplementary data include:

Localities, material and horizon

Methods

Systematic palaeontology

Supplementary description

Comparisons

Supplementary references

Supplementary Figure 1

Supplementary Figure 2

Supplementary Figure 3

Supplementary Figure 4

Supplementary Figure 5

Supplementary Figure 6

Supplementary Figure 7

Supplementary Figure 8

Supplementary Figure 9

Supplementary Figure 10

Supplementary Figure 11

Supplementary Figure 12

Supplementary Figure 13

**Localities, material and horizon**

The seed plant *Ganiopteris* gen. nov. was obtained from three localities (Lingtou, Tongling and Huashanling sections) in the northern part of Jiangxi Province of China (Fig. S1a-c). The fossils of *Ganiopteris* occur in three beds of the Lower Carboniferous Huashanling Formation at the Lingtou and Tongling sections (Fig. S1d), and in one bed of this formation at the Huashanling section (Fig. S1e). Most specimens come from the Lingtou section in Lidu Town and a few from the Tongling section in Wengang Town of Jinxian County of Nanchang District (Fig. 1a-j; Figs S1-9), and others from the Huashanling section in Dongjia Town of Fengcheng City of Yichun District (Fig. 1k-m; Figs S10-12). The plant was preserved as compressions or impressions in mudstone and siltstone. About one hundred and twenty specimens were collected, many of which had been prepared and photographed in the laboratory. Some specimens were photographed during the field work.

The early Carboniferous (Mississippian) sediments are mostly clastic rocks in Jiangxi Province, while the late Carboniferous (Pennsylvanian) sediments are nearly all marine carbonate rocks in this province (Zhou and Zhu 1978; Wu 1995; Wu and Zhu 2000; Chen et al. 2001; Ouyang et al. 2017). The Lower Carboniferous (Tournaisian) Huashanling Formation with clastic rocks is distributed in the north part of Jiangxi Province including Nanchang, Yichun, Yingtan, Fuzhou and Jingdezhen districts; this formation is underlain by the Upper Devonian (Famennian) stratum such as Wutong Formation or older sediments and is overlain by the Lower Carboniferous (Tournaisian-Visean) stratum such as the Zishan Formation or younger sediments (Fig. S1c; Wu 1995; Liu 1997; Wu and Zhu 2000; Ouyang et al. 2017). Huashanling Formation has characteristic plants such as *Adiantites*, *Calamites*, *Rhodea* and *Sphenopteris*, and Wutong Formation has *Leptophloeum rhombicum*. Zishan Formation with rich coal is widespread in Jiangxi Province and it contains *Neuropteris* and *Rhodea*.

At the Huashanling section near Longshan Reservoir of Dongjia Town, the type locality of the Huashanling Formation, this formation with a ca. 463 m thickness is underlain by the Neoproterozoic sediments and overlain by the Lower Carboniferous (Tournaisian) Zishan Formation (Ling et al. 2004). On the basis of occurrence and comparisons of plants including sphenopsids (*Hamatophyton verticillatum*), lycopsids (*Leptophloeum rhombicum*, *Lepidodendropsis hirmeri* and species of *Sublepidodendron*) and foliage (*Adiantites*, *Rhodea* and *Sphenopteris*), the Huashanling Formation here was regarded as Upper Devonian (upper Famennian) - Lower Carboniferous (Tournaisian) (Ling et al. 2004). In our field work, the Huashanling Formation at this section is measured to be only ca. 60 m thick with its lower and upper parts largely covered (Fig. S1e). From this formation, we collected plants probably assigned to the calamiteans, *Neuropteris*, *Pecopteris* and *Rhodea*, which more likely resemble an early Carboniferous rather than a Late Devonian assemblage. At our newly found sections of Lingtou and Tongling, the plant assemblage from the Huashanling Formation is the same as in the Huashanling section (Fig. S1d).

Fossil spores were obtained from two and five beds of the Huashanling Formation at the Lingtou and Huashanling sections, respectively (Fig. S1d, e, S13). The spores occur in the upper and basal beds (labelled as LT-2, LT-13) at the Lingtou section. Among the spore beds at the Huashanling Formation, the bed (HSL-10) gives rise to more genera. Pollen is completely absent in the strata investigated. Compared to the Late Paleozoic spores largely reported in South China (Ouyang et al. 2017), the spores from the Huashanling Formation include the Middle and/or Late Devonian *Retusotriletes semizonalis*, *Discernisporites macromanifestus*, *Apiculiretusispora commixta*, *Asperispora acuta*, *Grandispora macrospinosa*, the Devonian-early Carboniferous *Stenozonotriletes pumilus*, *Dictyotriletes varius*, *Aneurospora chinensis* and *Auroraspora pallida*, the Late Devonian-late Carboniferous *Discernisporites micromanifestus*, the (Early) Carboniferous *Grandispora minuta* and *Tripartites vetustus*, the early Carboniferous-Permian *Cyclogranisporites minutus* and *Lycospora* cf. *granulata*. These spores occur mainly near the boundary of the Devonian and Carboniferous (Ouyang et al. 2017). Such spore assemblage, megafossil plants and stratigraphic sequence thus date the Huashanling Formation as Lower Carboniferous (lower or lowermost Tournaisian).

**Methods**

Chisels and steel needles were used to prepare the very hard specimens. Serial dégagement was employed to reveal some fertile organs. All photographs were taken with a digital camera and Leica microscope, and the figures were completed with Photoshop CC, Adobe Illustrator CC and Adobe After Effects CC. Maceration of fossils, embedding and sections, and observation under SEM (Scanning Electron Microscope) failed to reveal the details of ovules.

**Systematic palaeontology**

Division Spermatophyta

Order Incertae sedis

Family Incertae sedis

*Ganiopteris phylla* gen. et sp. nov.

**Etymology**

The generic name from Jiangxi Province (this provincial name abbreviated as "Gan" in Chinese) where the plant was discovered, and from the Greek “pteron” (pinnule); the specific epithet from the Greek “phyllon” (leaf), referring to the megasporophyll with ovules.

**Holotype** **designated here**

PKUB21721a, b (part and counterpart housed in the Department of Geology, Peking University, Beijing) (Fig. 1a, b; Fig. S2a-d, k).

**Type locality and horizon**

Lingtou (28°15′12", 116°8′56"), Lidu, Jinxian, Jiangxi, China; Huashanling Formation, Lower Carboniferous (Tournaisian of Mississippian).

**Diagnosis**

Terminal strobilus with multiovulate fructifications or megasporophylls loosely and helically arranged on a central axis. Each megasporophyll consisting of a long pedicel and an obovate/flabellate lamina flanked by multiply dissected wings. Dorsiventral and bilaterally symmetrical megasporophyll bearing numerous ovules on abaxial side of lamina and showing many fan-shaped veins on adaxial side. Ovules generally ovoid in shape and wingless, and most probably with a micropyle.

**Supplementary description**

The strobili are 35.7-50.0 cm long and 6.4-15.0 cm wide, and their axes are up to 13.0 mm wide (Fig. S4a) and are attached at ca. 45° by pedicels of megasporophylls. The laminae of megasporophylls are 3.5-8.3 cm long and 2.4-7.5 cm wide. The wing lobes are 1.3-3.2 cm long and 1.0-2.6 mm at the maximum width. There are 18-26 wing lobes per megasporophyll. The pedicels of megasporophylls are 16.2-56.5 mm long and 2.0-4.7 mm wide, with the width slightly increased distally. There are 260-(320)-470 ovules per lamina of megasporophyll and their density is 20-(27)-62/cm^2^. The ovules are 1.3-(2.6)-3.8 mm long and 0.7-(1.4)-2.0 mm at the maximum width, and appear to lack obvious wings. Ovoid ovules are elongate and their distal taper is more apparent than their basal taper.

**Comparisons**

The gymnosperms with foliar-borne ovules (ovules attached to leaf laminae, pinnules or sporophylls) refer to the seed ferns (callistophytes, glossopterids and peltasperms), conifers, cordaites, cycads and gigantopterids (Taylor et al. 2009). Among them, the late Carboniferous – Early Permian callistophytes, late Carboniferous - Triassic peltasperms and Permian glossopterids, as in *Ganiopteris*, have ovules attached to the abaxial side of the leaf laminae. Nevertheless, the ovules of the callistophytes are solitary or only a few on the pinnules of the frond (Krings and Kerp 2000). Although the megasporophylls of the peltasperms may form a strobilus, they lack peripheral wing-like lobes and can be peltate, and the ovules are arranged in a ring or the number of ovules per leaf is only one to several (Kerp 1988; Taylor and Taylor 1992).

The glossopterids are characterized by usually planate polysperms (megasporophylls) with ovules borne on the abaxial surface or on abaxially/laterally recurved branchlets (McLoughlin and Prevec 2021). All types of glossopterid polysperms have marginal wing-like lobes (McLoughlin and Prevec 2019). *Ganiopteris* conforms to the glossopterids in the megasporophylls bearing abaxial ovules and marginal wing lobes. Individual polysperms of the glossopterids are subtended by a *Glossopteris* laminate vegetative leaf/bract to produce fertiligers, which are sometimes arranged in a loose strobilus (McLoughlin and Prevec 2019). The megasporophylls of *Ganiopteris* form a loose strobilus, but they lack a subtending laminate vegetative leaf. This presents a great difference from the glossopterids and likely has evolutionary significance in a palaeogeographic or climatic background. The megasporophylls of *Ganiopteris* occur in helices, while the arrangement pattern of the fertiligers of most glossopterids is rarely known due to poor preservation.

Among Glossopteridales, the Dictyopteridiaceae and Lidgettoniaceae fit well with *Ganiopteris* in the megasporophylls with evident lamina (receptacle) fringed by closely arranged striate wing lobes. *Ganiopteris* closely resembles the Dictyopteridiaceae, especially *Karingbalia* (McLoughlin 2016), *Ottokaria* (White 1978), *Plumsteadia* (Rigby 1978) and *Scutum* (Plumstead 1952), in the scutiform megasporophyll with a large lamina bearing numerous abaxial ovules and multiple wing lobes. The ovule length (1.3-(2.6)-3.8 mm) and ovule number per megasporophyll (ca. 320) of *Ganiopteris* approximately fall within those of the Dictyopteridiaceae (0.5-4.0 mm, 50-400, McLoughlin and Prevec 2021), respectively. The megasporophylls of the Lidgettoniaceae, as in *Ganiopteris*, have curved wings to partly surround the ovules, but they are small and cupule-like.

**Supplementary references**

1. Kerp, J.H., 1988. Aspects of Permian palaeobotany and palynology. X. The West- and Central European species of the genus *Autunia* Krasser emend. Kerp (Peltaspermaceae) and the form-genus *Rhachiphyllum* Kerp (callipterid foliage). Review of Palaeobotany and Palynology, 54, 249–360.
2. Krings, M., Kerp, H., 2000. A contribution to the knowledge of the pteridosperm genera *Pseudomariopteris* Danzé-Corsin nov. emend. and *Helenopteris* nov. gen. Review of Palaeobotany and Palynology, 111, 145–195.
3. Ling, L.-H., Lou, F.-S., Feng, Y., Liu, C.-G., 2004. The Devonian lithostratigraphic classification and correlation of Jiangxi Province. Journal of Stratigraphy, 28, 126–136 (in Chinese with English abstract).
4. Liu, Y.-G., 1997. Stratigraphy (Lithostratic) of Jiangxi Province. China University of Geosciences Press. Wuhan.
5. McLoughlin, S., 2016. A new genus of glossopterid fructifications from the Artinskian to Changhsingian of eastern Australia. Ameghiniana, 53, 586–598.
6. McLoughlin, S., Prevec, R., 2019. The architecture of Permian glossopterid ovuliferous reproductive organs. Alcheringa, 43, 480–510.
7. Ouyang, S., Lu, L.-C., Zhu, H.-C., Liu, F., 2017. The Late Paleozoic spores and pollen of China. University of Science and Technology of China Press, Hefei.
8. Plumstead, E.P., 1952. Description of two new genera and six new species of fructifications borne on *Glossopteris* leaves. Transactions of the Geological Society of South Africa, 55, 281–320.
9. Rigby, J.F., 1978. Permian glossopterid and other cycadopsid fructifications from Queensland. Geological Survey of Queensland Publication 367, Palaeontological Paper 41, 1–21.
10. Taylor, E.L., Taylor, T.N., 1992. Reproductive biology of the Permian Glossopteridales and their suggested relationship to flowering plants. Proceedings of the National Academy of Sciences of the United States of America, 89, 11495–11497.
11. White, M.E., 1978. Reproductive structures of the Glossopteridales in the plant fossil collection of the Australian Museum. Records of the Australian Museum, 31, 473–505.
12. Wu, X.-Y., 1995. Carboniferous floras, in: Li, X.-X., Zhou, Z.-Y., Cai, C.-Y., Sun, G., Ouyang, S., Deng, L.-H. (Eds.), Fossil floras of China through the geological ages. Guangdong Science and Technology Press, Guangzhou, pp. 78–126.
13. Wu, X.-Y., Zhu, H.-C., 2000. The Non-marine Carboniferous, in: Nanjing Institute of Geology and Palaeontology, Chinese Academy of Sciences (Eds.), Stratigraphical Studies in China (1979–1999). University of Science and Technology of China Press, Hefei, pp. 165–187.
14. Zhou, D.-C., Zhu, Z.-G., 1978. An overview of the Devonian in Jiangxi Province, in: Institute of Mineral Resources, Chinese Academy of Geological Sciences (Eds.), Proceedings of the Conference on the Devonian System in South China. Geological Publishing House, Beijing, pp. 184–188.

**Fig. S1.** Map showing the locality and stratigraphy of glossopterid-like plant *Ganiopteris phylla* gen. et sp. nov. **a,** Jiangxi Province, where the fossil plant was discovered. **b,** Red rectangle area within which the fossil plant occurs. **c,** Distribution of early Carboniferous deposits of the Huashanling Formation (C_1_h, purple oblique lines) and Zishan Formation (C_1_z, green area), and localities of the Lingtou, Tongling and Huashanling sections (red stars). **d, e,** Stratigraphic columns of the Huashanling Formation at the Lingtou and Tongling sections, and at the Huashanling section, respectively, showing lithology and beds of plants including *G. phylla* and spores (for details of spores, see Fig. S13). Scale bars for spores: 10 μm.

**Fig. S2.** Strobilus with megasporophylls of *Ganiopteris phylla* from the Lingtou section. **a-d,** Serial dégagement of strobilus (PKUB21721a). **b, d,** The same as in Fig. 1a, b, respectively. **e,** Enlargement of arrowed part in a, showing pedicel scar (arrow) and pedicels. **f,** Enlarged part in b (arrow 1) showing strobilar axis (arrow 2), pedicel (arrow 1) and pedicel scar (arrow 3). **g,** Enlarged part in b (arrow 2), lateral view of sporophyll wings. **h,** Enlarged part in b (arrow 3), abaxial view of sporophyll lamina. **i,** Enlarged sporophyll in b (arrow 4). **j,** Enlarged sporophyll in d (upper arrow). **k,** Counterpart of upper part of strobilus in d (PKUB21721b). **l, m,** Enlarged parts in d (lower arrow), c (arrow), adaxial and abaxial views of sporophylls, respectively. **n, o,** Enlargement of rectangular parts in i and m, respectively, showing abaxial ovules on laminae. Scale bars: 5 cm (a-d), 1 cm (e-m), 5 mm (n, o).

**Fig. S3.** Strobilus with megasporophylls of *Ganiopteris phylla* from the Lingtou section. **a,** Strobilus before dégagement (PKUB26002a). **b,** Enlargement after removal of arrowed part in a. **c, d,** Serial dégagement of strobilus in a. **d,** The same as in Fig. 1c. **e,** Enlargement of d (upper arrow), showing attachment of three pedicels. **f,** Enlarged sporophyll in c (arrow 1). **g,** Enlarged part in d (lower arrow). **h,** Enlarged part in c (arrow 2), vascular strand in pedicel. **i,** Counterpart of sporophyll in j (PKUB26002b). **j,** Enlarged sporophyll in c (arrow 3) after dégagement, arrow showing a pedicel. **k,** Enlarged sporophyll in c (arrow 4). Scale bars: 2 cm (a, c, d), 1 cm (b, e-k).

**Fig. S4.** Strobili of *Ganiopteris phylla* from the Lingtou section. **a,** A long axis with a terminal strobilus (PKUB26005). **b-e,** Enlargement of four parts in a (b, c, d, e), respectively. **b-d,** Strobilar axis with sporophylls. e, Axis lacking sporophylls. **f,** Another strobilus (PKUB26006). **g, h,** Enlarged sporophylls in f (upper and lower arrows), respectively. Scale bars: 10 cm (a), 2 cm (b-f), 1 cm (g, h).

**Fig. S5.** Strobilus of *Ganiopteris phylla* from the Lingtou section. **a,** A strobilus with helically arranged megasporophylls (PKUB26007a). **b,** Counterpart of strobilus in a (PKUB26007b). **c,** Enlarged part in b (arrow), strobilar axis with sporophylls. **d,** Two enlarged megasporophylls from a (arrows 1 and 2). **e,** Enlarged part in d (upper rectangle). **f, g,** Enlarged parts in a (arrows 3 and 4), respectively. **h,** Enlarged part in g (rectangle). **i,** Enlarged part in d (lower rectangle). **j, k,** Enlarged parts in i (arrow) and h (arrow), respectively. Scale bars: 2 cm (a, b), 1 cm (c, d, g), 5 mm (f), 2 mm (e, h, i), 1 mm (j, k).

**Fig. S6.** Strobili and megasporophylls of *Ganiopteris phylla* from the Lingtou section. **a, b,** Part and counterpart of a short strobilus, arrow showing sporophyll in adaxial view (PKUB26008a, PKUB26008b). **c,** One short strobilus (PKUB26009). **d,** A sporophyll associated with vegetative foliage (PKUB26010). **e, f,** Part and counterpart of short strobili (PKUB26011a, PKUB26011b). **g-i,** Three sporophylls in adaxial view (PKUB26017, PKUB26018, PKUB26019). Scale bars: 1 cm (a-i).

**Fig. S7.** Strobilus and megasporophylls of *Ganiopteris phylla* from the Lingtou section. **a, b,** A strobilus viewed from two opposite sides (PKUB26012). **c, d,** Enlarged parts in a (left and right arrows), respectively. **d,** Lateral view of sporophyll with wings. **e,** Abaxial view of sporophyll in d. **f, g,** Enlarged parts in b (upper and lower arrows), respectively. **h, j, k,** Lateral view of sporophyll with wings (PKUB26020, PKUB26021, PKUB26022). **i,** Abaxial view of sporophyll in h. Scale bars: 2 cm (a, b), 1 cm (c-k).

**Fig. S8.** Strobilus of *Ganiopteris phylla* from the Lingtou section. **a, b,** Part and counterpart of a strobilus (PKUB26013a, PKUB26013b). **c, d, f,** Enlarged megasporophylls in a (upper and lower arrows) and b (arrow 1), respectively. **e,** Counterpart of sporophyll in d (PKUB26013c). **g-i,** Enlarged sporophylls in b (arrows 2-4), respectively. **j,** Dégagement of sporophyll in i. Scale bars: 5 cm (a, b), 2 cm (d, e), 1 cm (c, f-j).

**Fig. S9**. Strobilus and megasporophyll of *Ganiopteris phylla* from the Tongling section. **a, b,** Part and counterpart of strobilus with sporophylls in adaxial view. **c, d,** Enlarged parts in a (arrow) and b (arrow), respectively. Arrow in d indicating enlarged part in f. **e, f,** Sporophylls in adaxial view (PKUB26023, PKUB26024). Scale bars: 5 cm (a, b), 2 cm (c, d), 1 cm (e, f).

**Fig. S10.** Strobilus and megasporophylls of *Ganiopteris phylla* from the Huashanling section. **a-c,** Serial dégagement of a strobilus (PKUB26014). **d,** Enlarged sporophyll in b (arrow 1). **e,** Enlarged strobilar axis in c, attachment of two pedicels. **f, g,** Enlarged sporophylls in b (arrows 2 and 3), respectively. **h,** Enlarged part in d (rectangle), showing ovules on abaxial side of sporophyll. **i-k,** Abaxial view of sporophylls with pedicels (PKUB26025, PKUB26026). Scale bars: 2 cm (a-c), 1 cm (d-g, i-k), 5 mm (h).

**Fig. S11.** Megasporophylls of *Ganiopteris phylla* from the Huashanling section. **a, b,** Two sporophylls in abaxial view. The same as in Fig. 1k, l, respectively (PKUB26015, PKUB26016). **c-e, i,** Enlarged parts in a (rectangles 1-4), respectively, showing ovules. **f,** Enlarged ovules in b (arrow). **g,** Enlarged part in c (rectangle). **h,** Enlarged ovules in d (arrow). Scale bars: 1 cm (a, b), 5 mm (c), 2 mm (d-g, i), 1 mm (h).

**Fig. S12.** Megasporophylls of *Ganiopteris phylla* from the Huashanling section. **a-e,** Five sporophylls in abaxial view (PKUB26027, PKUB26028, PKUB26029, PKUB26030, PKUB26031). **f,** Enlarged part in c (rectangle). **g, h,** Enlarged parts in e (lower and upper rectangles), respectively. **i, j,** Enlarged ovules in f (arrow) and g (arrow), respectively. Scale bars: 1 cm (a-e), 5 mm (f-h), 2 mm (i, j).

**Fig. S13.** Representative spores from the Huashanling Formation at the Lingtou and Huashanling sections. **a,** *Retusotriletes semizonalis* (D2-D3, LT-2). **b,** *Discernisporites macromanifestus* (D2-D3, HSL-10). **c, d,** *Apiculiretusispora commixta* (D3, LT-13). **e-g,** *Asperispora acuta* (D3, LT-2, LT-13, HSL-10, respectively). **h,** *Grandispora macrospinosa* (D3, HSL-10). **i,** *Stenozonotriletes pumilus* (D1-C1, HSL-10). **j, k,** *Dictyotriletes varius* (D2-C1, HSL-4, HSL-3, respectively). **l,** *Aneurospora chinensis* (D3-C1t, HSL-10). **m,** *Auroraspora pallida* (D3-C1t, HSL-8). **n,** *Discernisporites micromanifestus* (D3-C2, HSL-10). **o,** *Grandispora minuta* (C1t, HSL-10). **p,** *Tripartites vetustus* (C1-C2, HSL-12). **q-s,** *Cyclogranisporites minutus* (C1-P1, LT-13, LT-2, HSL-10, respectively). **t,** *Lycospora* cf. *granulata* (C1-P2, HSL-10). D1: Early Devonian, D2: Middle Devonian, D3: Late Devonian, C1: early Carboniferous, C1t: early Carboniferous (Tournaisian), C2: late Carboniferous, P1: Early Permian, P2: Late Permian. LT-2, LT-13: beds 2 and 13 of the Lingtou section, respectively. HSL-3, HSL-4, HSL-10, HSL-12: beds 3, 4, 10, 12 of the Huashanling section, respectively. Scale bars: 10 μm.
